# Supplementary material for: Tailor-Made Immunochromatographic Test for the Detection of Multiple 17α-Methylated Anabolics in Dietary Supplements
Source: Foods. 2021 Apr 1;10(4):741. doi: 10.3390/foods10040741 (PMC8065520; doi:10.3390/foods10040741)
Supplement: Supplementary file 1 [file foods-10-00741-s001.pdf]

# Supplementary Material

## Tailor-Made Immunochromatographic Test for the Detection of Multiple 17$\alpha$-Methylated Anabolics in Dietary Supplements

**Barbora Holubová [1,*], Pavla Kubešová [1], Lukáš Huml [2], Miroslav Vlach [1], Oldřich Lapčík [2], Michal Jurášek [2] and Ladislav Fukal [1]**

University of Chemistry and Technology Prague, CZ-166 28 Prague, Czech Republic, 1Department of Biochemistry and Microbiology, 2Department of Chemistry of Natural Compounds

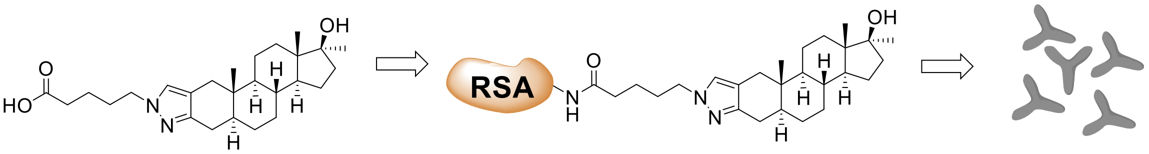


ST-3 RSA/ST-3 RAb 212

**Figure 1.** Chemical structures of stanazolol hapten (**ST-3**) and its conjugate with RSA (**RSA/ST-3**). By immunization of rabbit, group selective polyclonal antibodies (**RAb 212**) were obtained[1].

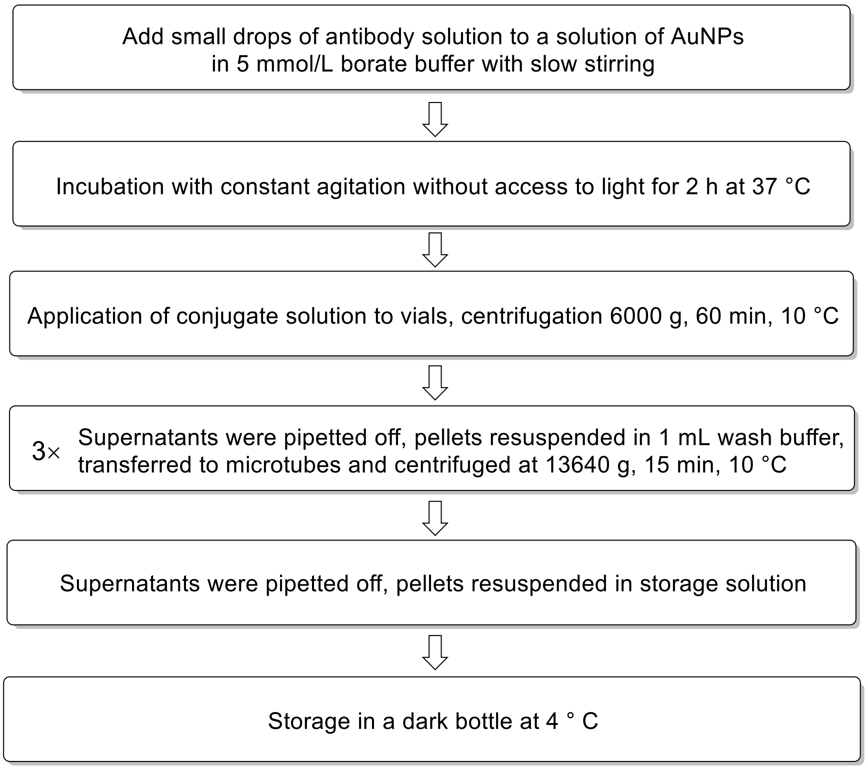


**Figure S2.** Procedure for the preparation of colloidal RAb 212/AuNPs solution.

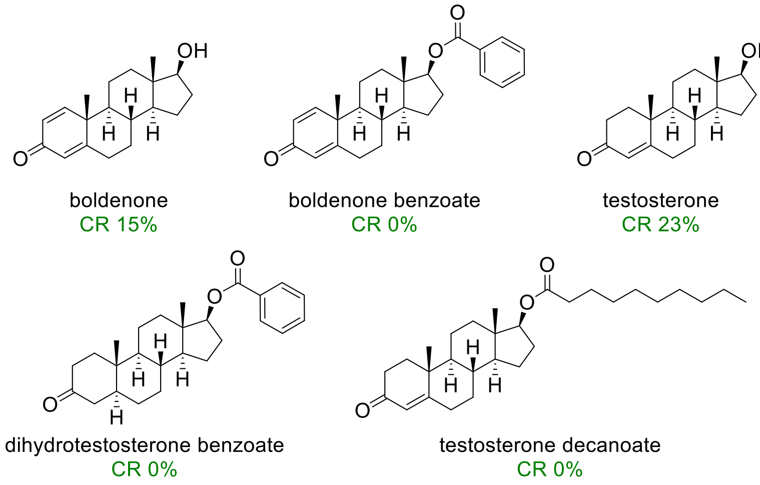


**Figure S3.** Tested steroids with low CR.

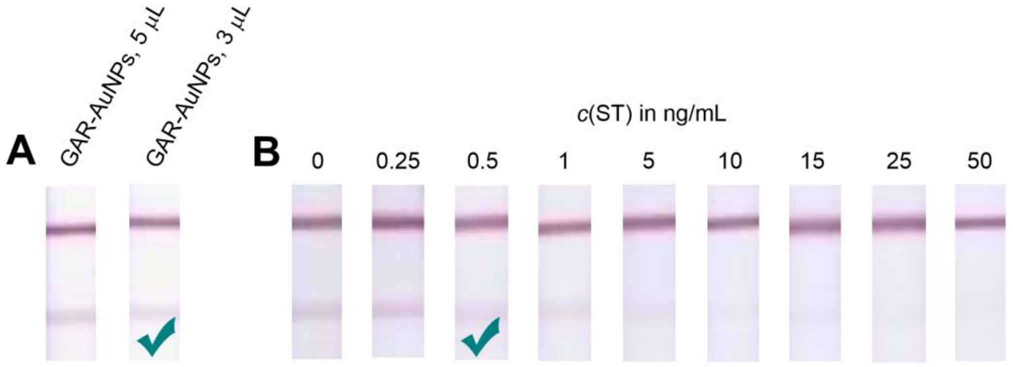


**Figure S4.** Selection of the applied amount of GAR-AuNPs on panel A and the calibration series for indirect open format (3 µL GAR-AuNPs were applied) on panel B.

**Table S1.** Original and newly selected test materials for immunochromatographic test (closed format), including criteria in which the individual materials differed.

| **Membrane** | | | **Manufacturer / supplier** |
|---|---|---|---|
| **designation** | **pore size [µm]** | **note** | |
| AE 98 | 5 | selected | Whatman GmbH, Dassel, Germany |
| AE 99 | 8 | tested | Whatman GmbH, Dassel, Germany |
| AE 100 | 12 | tested | Whatman GmbH, Dassel, Germany |
| PRIMA 85 | 8 | tested | Whatman GmbH, Dassel, Germany |
| PRIMA 125 | 12 | tested | Whatman GmbH, Dassel, Germany |
| FF80HP | not specified | tested | Whatman GmbH, Dassel, Germany |
| FF120HP | not specified | tested | Whatman GmbH, Dassel, Germany |
| HFB13504 | 8 | tested | Millipore Corp., Massachusetts, USA |
| HFB135UB | 8 | tested | Millipore Corp., Massachusetts, USA |
| **Membrane pad** | | | |
| **designation** | **material** | **note** | |
| HF000MC100 | laminate | selected | Millipore Corp., Massachusetts, USA |
| ARcare® 8192 | plastic | tested | Adhesive Research, Limerick, Ireland |
| **Sample pad** | | | |
| **designation** | **material** | **note** | |
| CFSP 173000 | cellulose | original | Millipore Corp., Massachusetts, USA |
| CFSP 223000 | cellulose | tested | Millipore Corp., Massachusetts, USA |
| FUSION 5 | glass and synthetic material | tested | Whatman GmbH, Dassel, Germany |
| Grade 121 | glass and synthetic material | tested | Ahlstrom, Kotka, Finland |
| Grade 142 | glass and synthetic material | tested | Ahlstrom, Kotka, Finland |
| Grade 1281 | cotton | selected | Ahlstrom, Kotka, Finland |
| **Conjugation pad** | | | |
| **designation** | **material** | **note** | |
| GFCP 103000 | glass fibers | original | Millipore Corp., Massachusetts, USA |
| CFDX 103000 | glass fibers | tested | Millipore Corp., Massachusetts, USA |
| Grade 6613 | polyester | tested | Ahlstrom, Kotka, Finland |
| Grade 6615 | polyester | selected | Ahlstrom, Kotka, Finland |
| Grade 8950 | glass fibers | tested | Ahlstrom, Kotka, Finland |
| Grade 8951 | glass fibers | tested | Ahlstrom, Kotka, Finland |
| **Absorption pad** | | | |
| **designation** | **material / thickness [mm]** | **note** | |
| CFSP 173000 | cellulose / 0.83 | original | Millipore Corp., Massachusetts, USA |
| CFSP 223000 | cellulose / 0.83 | tested | Millipore Corp., Massachusetts, USA |
| Grade 222 | cotton / 0.83 | tested | Ahlstrom, Kotka, Finland |
| Grade 237 | cotton / 0.42 | tested | Ahlstrom, Kotka, Finland |
| Grade 238 | cotton / 0.34 | tested | Ahlstrom, Kotka, Finland |
| Grade 319 | cotton / 0.48 | tested | Ahlstrom, Kotka, Finland |
| Grade 320 | cotton / 2.48 | selected | Ahlstrom, Kotka, Finland |
| Grade 601 | cotton / 0.19 | tested | Ahlstrom, Kotka, Finland |

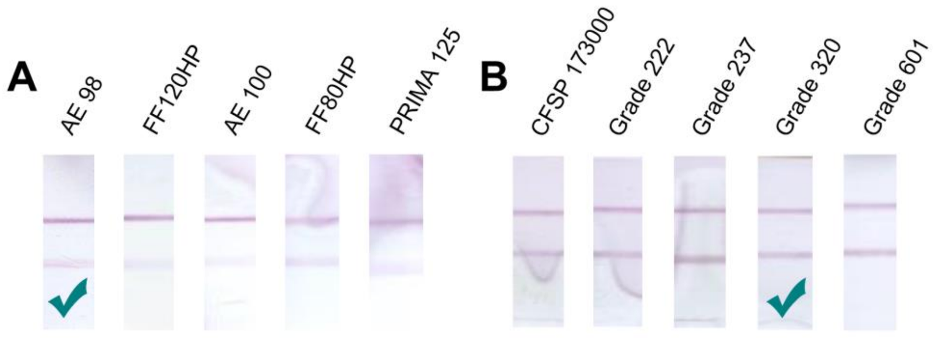


**Figure S5.** Some results of tested membranes (panel A) and absorbent pads (panel B) for indirect closed format.

**Table S2.** Summary of tested drying and reaction buffers

| **Drying buffers** |
|---|
| 0.2 mol/L borate buffer-0.1% BSA-3% trehalose-1% Tween 20 |
| 0.1 mol/L borate buffer-0.1% BSA-3% trehalose-1% Tween 20 |
| 0.2 mol/L borate buffer-0.1% BSA-1% Tween 20 |
| 0.2 mol/L borate buffer-1% BSA-3% trehalose-1% Tween 20 |
| 0.2 mol/L borate buffer-0.1% BSA-3% trehalose-0.5% Tween 20 |
| 0.1 mol/L borate buffer-1% BSA-3% trehalose-1% Tween 20 |
| 0.1 mol/L borate buffer-0.1% BSA-3% trehalose-0.5% Tween 20 |
| **Reaction buffers** |
| 0.1 mol/L borate buffer-1% BSA-1% PEG- 1%Tween 20 |
| 0.2 mol/L borate buffer-1% BSA-1% PEG- 1%Tween 20 |
| 0.1 mol/L borate buffer-1% PEG-1% Tween 20 |
| 0.1 mol/L borate buffer-1% BSA-1% Tween 20 |
| 0.1 mol/L borate buffer-1% BSA-1% PEG |
| 0.1 mol/L borate buffer-0.5% BSA-1% PEG- 1%Tween 20 |
| 0.1 mol/L borate buffer-2% BSA-1% PEG- 1%Tween 20 |
| 0.1 mol/L borate buffer-1% BSA-0.5% PEG- 1%Tween 20 |
| 0.1 mol/L borate buffer-1% BSA-2% PEG- 1%Tween 20 |
| 0.1 mol/L borate buffer-1% BSA-1% PEG- 0.5%Tween 20 |
| 0.1 mol/L borate buffer-1% BSA-1% PEG- 2%Tween 20 |

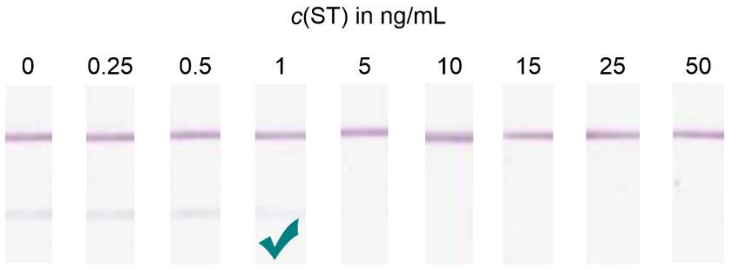


**Figure S6.** Calibration series for indirect closed format (3 µL GAR-AuNPs were applied).

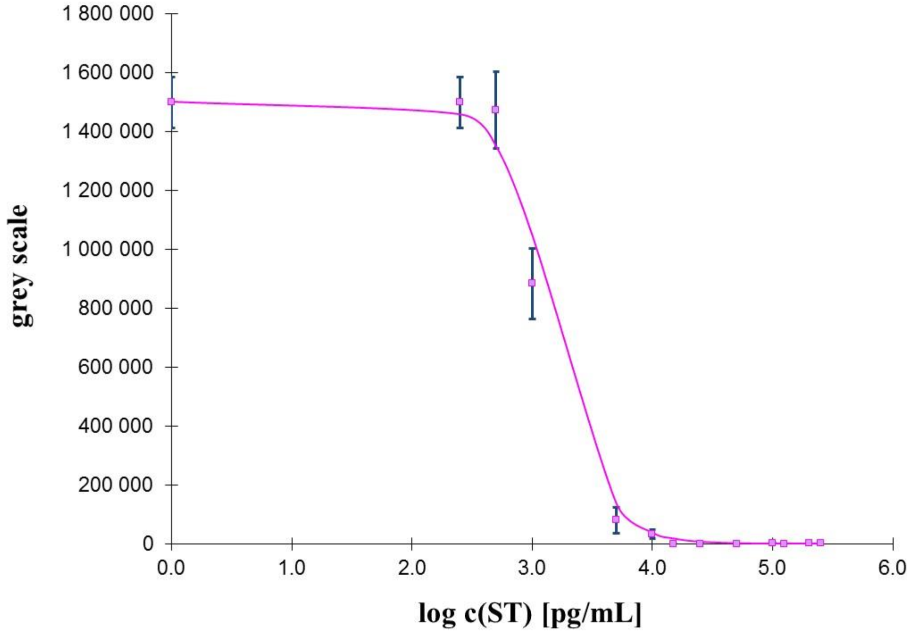


**Figure S7.** Calibration curve for indirect ICT format. The error bars correspond to the standard deviations of the data points (n = 3)

**Table S3.** Characteristics of ST standard curves for indirect and direct format (data from 3 analyses).

| ICT format | Linear working range[a] (ng/mL) | Limit of detection ± SD[b] (ng/mL) | $IC_{50}$ ± SD[b] (ng/mL) |
|---|---|---|---|
| direct | 0.75 – 3.13 | 0.34 ± 0.09 | 1.4 ± 0.4 |
| indirect | 0.41 – 3.8 | 0.42 ± 0.12 | 1.6 ± 0.4 |

[a] Expressed as the concentration range causing 20-80% inhibition of the maximal assay signal.

[b] Standard deviation.

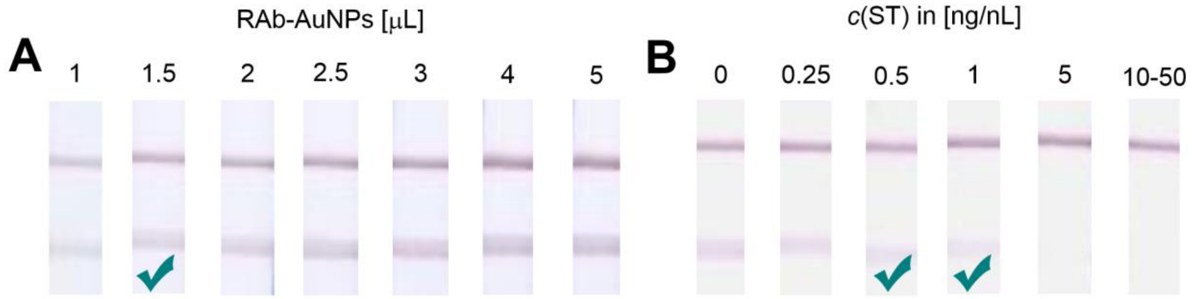


**Figure S8.** Selection of the applied amount of RAb-AuNPs on panel A and the calibration series for direct open format (1.5 µL RAb-AuNPs were applied) on panel B.

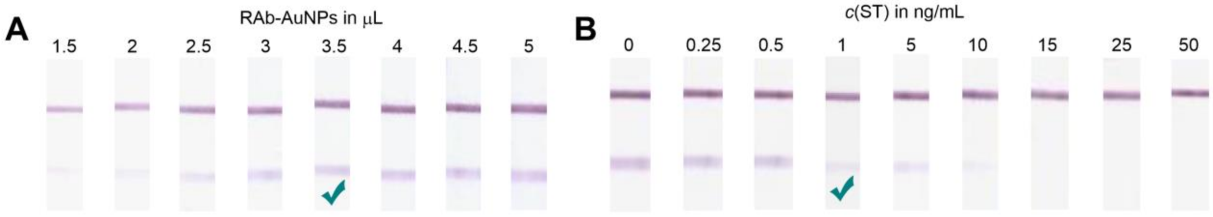


**Figure S9.** Tested amounts of applied RAb-AuNPs for closed format (panel A) and calibration series for direct closed format (3.5 µL of RAb-AuNPs was applied; panel B).

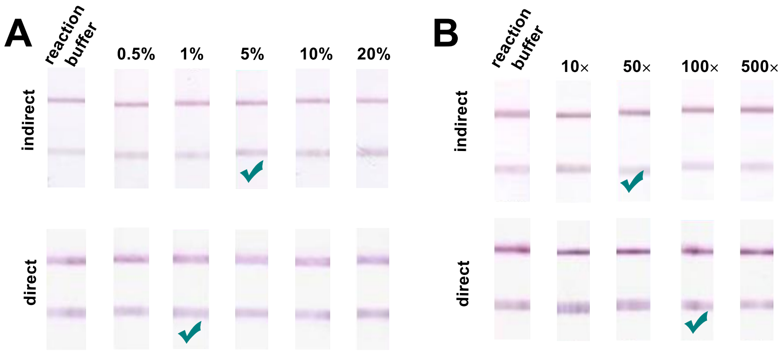


**Figure S10**. Testing of extraction solvent (panel A) and matrix effect (panel B) on the functionality of RAb 212 antibody.

**Table S4.** Matrices used for contamination with AAS

| Food supplement | | | Manufacturer / supplier |
|---|---|---|---|
| **Matrix No.** | **designation** | **content** | **-** |
| **1** | 100% whey protein | ultrafiltered whey protein | SCITEC NUTRITION, Orlando, FL, USA |
| **2** | Gain Tech | weight gainer | ALL STARS, Peißenberg, DE |
| **3** | Magnus | weight gainer | PENCO, Řevnice, CZ |
| **4** | Compress B.I.G. | instant gainer | NUTREND D.S., Olomouc, CZ |
| **5** | TRUE-MASS | mass gainer | Bio-Engineered Supplements & Nutrition, Boca Raton, FL, USA |
| **6** | NITRIX | Vaso-muscular volumizer | Bio-Engineered Supplements & Nutrition, Boca Raton, FL, USA |
| **7** | AMIX EGG AMINO 6000 | fat-free amino formula | Large Life Ltd., Manchester, UK |
| **8** | METHOXY-7-TEST | anabolic support complex | PhD Nutrition Ltd., Hull, UK |
| **9** | BCAA Complex 2200 | branched-chain amino acids | Dymatize Enterprises Inc., Dallas, TX, USA |
| **10** | BCAA X | BCAA muscle preservation system | SCITEC NUTRITION, Orlando, FL, USA |

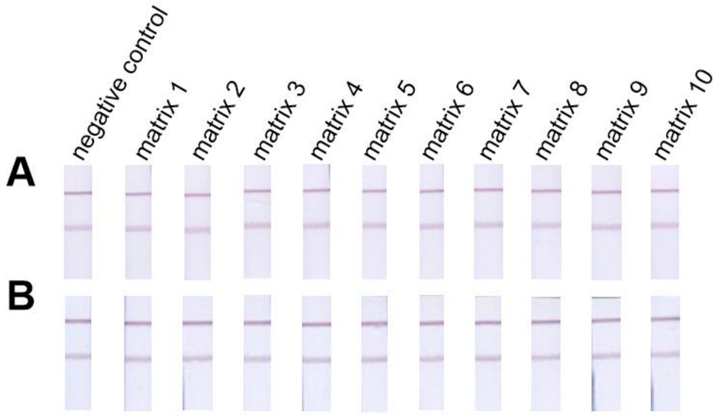


**Figure S11**. Tested effect of selected matrices in indirect (panel A) and direct format (panel B) opened ICT format. The designation of the matrices is given above in Table S3.

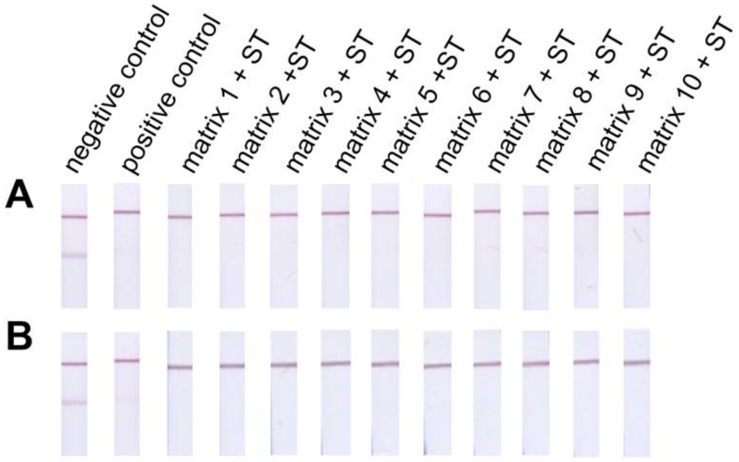


**Figure S12.** Tested selected matrices with the addition of ST with 250× dilution; indirect (panel A) and direct (panel B) opened ICT format.

## References

1. Huml, L.; Havlová, D.; Longin, O.; Staňková, E.; Holubová, B.; Kuchař, M.; Prokudina, E.; Rottnerová, Z.; Zimmermann, T.; Drašar, P.; Lapčík, O.; Jurášek, M., Stanazolol derived ELISA as a sensitive forensic tool for the detection of multiple 17$\alpha$-methylated anabolics. *Steroids* **2020,** *155*, 108550.
